# Spaceflight-Associated Changes of snoRNAs in Peripheral Blood Mononuclear Cells and Plasma Exosomes—A Pilot Study

**DOI:** 10.3389/fcvm.2022.886689

**Published:** 2022-06-24

**Authors:** Amit Kumar Rai, K. Shanmugha Rajan, Malik Bisserier, Agnieszka Brojakowska, Aimy Sebastian, Angela C. Evans, Matthew A. Coleman, Paul J. Mills, Arsen Arakelyan, Shizuka Uchida, Lahouaria Hadri, David A. Goukassian, Venkata Naga Srikanth Garikipati

**Affiliations:** ^1^Department of Emergency Medicine, The Ohio State University Wexner Medical Center, Columbus, OH, United States; ^2^Department of Chemical and Structural Biology, Weizmann Institute of Science, Rehovot, Israel; ^3^Cardiovascular Research Institute, Icahn School of Medicine at Mount Sinai, New York, NY, United States; ^4^Physical and Life Sciences Directorate, Lawrence Livermore National Laboratory, Livermore, CA, United States; ^5^Department of Radiation Oncology, University of California, Davis, Sacramento, CA, United States; ^6^Center of Excellence for Research and Training in Integrative Health, University of California, San Diego, La Jolla, CA, United States; ^7^Group of Bioinformatics, Institute of Molecular Biology, National Academy of Sciences of the Republic of Armenia, Yerevan, Armenia; ^8^Center for RNA Medicine, Department of Clinical Medicine, Aalborg University, Copenhagen, Denmark; ^9^Dorothy M. Davis Heart Lung and Research Institute, The Ohio State University Wexner Medical Center, Columbus, OH, United States

**Keywords:** snoRNA, biomarker, astronaut, extracellular vesicles, peripheral blood—mononuclear cells

## Abstract

During spaceflight, astronauts are exposed to various physiological and psychological stressors that have been associated with adverse health effects. Therefore, there is an unmet need to develop novel diagnostic tools to predict early alterations in astronauts’ health. Small nucleolar RNA (snoRNA) is a type of short non-coding RNA (60–300 nucleotides) known to guide 2′-O-methylation (Nm) or pseudouridine (ψ) of ribosomal RNA (rRNA), small nuclear RNA (snRNA), or messenger RNA (mRNA). Emerging evidence suggests that dysregulated snoRNAs may be key players in regulating fundamental cellular mechanisms and in the pathogenesis of cancer, heart, and neurological disease. Therefore, we sought to determine whether the spaceflight-induced snoRNA changes in astronaut’s peripheral blood (PB) plasma extracellular vesicles (PB-EV) and peripheral blood mononuclear cells (PBMCs). Using unbiased small RNA sequencing (sRNAseq), we evaluated changes in PB-EV snoRNA content isolated from astronauts (*n* = 5/group) who underwent median 12-day long Shuttle missions between 1998 and 2001. Using stringent cutoff (fold change > 2 or log_2_-fold change >1, FDR < 0.05), we detected 21 down-and 9—up-regulated snoRNAs in PB-EVs 3 days after return (R + 3) compared to 10 days before launch (L-10). qPCR validation revealed that SNORA74A was significantly down-regulated at R + 3 compared to L-10. We next determined snoRNA expression levels in astronauts’ PBMCs at R + 3 and L-10 (*n* = 6/group). qPCR analysis further confirmed a significant increase in SNORA19 and SNORA47 in astronauts’ PBMCs at R + 3 compared to L-10. Notably, many downregulated snoRNA-guided rRNA modifications, including four Nms and five ψs. Our findings revealed that spaceflight induced changes in PB-EV and PBMCs snoRNA expression, thus suggesting snoRNAs may serve as potential novel biomarkers for monitoring astronauts’ health.

## Introduction

During spaceflight, astronauts are exposed to various physiological and psychological stressors, such as microgravity, sleep deprivation, isolation, confinement, and high ionizing radiation (1, 2). Despite the risk mitigation plans currently in place, it is anticipated that these stressors will have more prominent adverse health effects during deep-space exploration missions, including but not limited to loss of muscle mass, immune cell dysfunction, neurodegeneration, cancers, and cardiovascular diseases (CVDs). Therefore, space travel-associated adverse health conditions remain a considerable concern for astronauts (3, 4). Consequently, there is an unmet need to develop novel diagnostic tools to predict early alterations in astronauts’ health.

Extracellular vesicles (EVs) are secreted from most cells and are enriched in different non-coding RNA (ncRNA) species which serve as significant drivers of intracellular communication in physiology and pathological conditions (5–7). The mechanism of action of EV-mediated molecular signaling primarily includes the transfer of the exosomal cargo (e.g., parent cell-specific ncRNAs) to target cells (8), resulting in the dysregulation of various biological processes (9). Several RNA species, including mRNA, miRNA, lncRNA and other ncRNAs, are identified in the EV cargo, of which miRNA is the most characterized (5, 9–12). The long-standing assumption that snoRNAs function as housekeeping genes has been challenged in recent years, unveiling their likely utility as predictive biomarkers for a wide variety of diseases (13–15). Small nucleolar RNAs (snoRNAs) are a class of short ncRNAs (60–300 nucleotides) usually located in the nucleus of eukaryotic organisms. Typically, most snoRNAs are generated co-transcriptionally with a host gene, followed by splicing, branching of the intron lariat, and exonucleolytic digestion in the nucleoplasm (16). snoRNA carry either C/D or H/ACA motifs. C/D snoRNAs typically contain C/D box motifs (C: RUGAUGA and D: CUGA) that recruit proteins vital to forming the 2′-O-methyltransferase complexes, including fibrillarin in eukaryotes (NOP1 in yeast). H/ACA snoRNA contain H and ACA motifs (H: ANANNA and ACA) and participate in pseudouridylation by recruiting a pseudouridine synthase enzyme dyskerin in eukaryotes (Cbf5 in yeast) to the target RNA (17, 18). snoRNA are known to guide 2′-O-methylation (Nm) or pseudouridine (ψ) on rRNA, snRNA, or mRNA (19). Over recent years, snoRNAs have gained increasing interest among the scientific community. Emerging evidence suggests that dysregulation of snoRNAs may be key players in regulating fundamental cellular mechanisms and the pathogenesis of diabetes, cancer, CVDs, and neurological disease; thus, snoRNAs may prove to be potential biomarkers for detecting human diseases (20–24). The present study aimed to assess the effects of spaceflight on astronauts, peripheral blood (PB) plasma EVs (PB-EVs) snoRNA content and describe the differential expression of spaceflight-induced snoRNAs in astronaut peripheral blood mononuclear cells (PBMCs).

## Materials and Methods

### Astronaut Samples

This study was determined to be a non-human study by NASA’s Institutional Review Board (IRB) and the Icahn School of Medicine at Mount Sinai’s IRB (STYDY00000075 and HSM19-00367, respectively). Samples were collected as part of a NASA flight study (25). All participants provided written informed consent to have blood stored and used for retrospective analysis. PB was sampled 10 days before launch (L-10) and 3 days after return (R + 3) from five astronauts (S12, S30, S31, S34, and S39) who flew short (median 12 day long) Shuttle missions between 1998 and 2001. De-identified blood samples were stored at −80°C until use.

### Thrombin Plasma Preparation for Extracellular Vesicle Precipitation

Exosomes were isolated from blood plasma samples of five astronauts (S12, S30, S31, S34, and S39) at L-10 and R + 3 using the ExoQuick Plasma prep and Exosome precipitation kit (Cat # EXOQ5TM, System Biosciences, CA, United States), as previously described (26). Briefly, thrombin was added to the plasma and incubated for 5 min at room temperature (RT). Next, samples were centrifuged at 10,000 rpm for 5 min, and the supernatant was incubated with the exosome precipitation solution for 30 min at 4°C. Next, samples were centrifuged for 30 min at 1,500 × *g* at 4°C. Finally, the supernatant was aspirated, and the beige-colored pellet was dissolved in 100 μl of sterile 1× PBS.

### RNA Isolation

According to the manufacturer’s protocol, total RNA was extracted from astronaut-derived exosomes using PB collected at L-10 and R + 3 using the RNeasy Mini Kit (Qiagen). High-quality RNA was isolated using RNeasy Mini spin columns and eluted using molecular-grade water (RNAse/DNAse free). All samples first undergo quality control assessment to ensure successful library preparation and sequencing. RNA sample quality was assessed by NanoDrop and Agilent 2100 BioAnalyzer. Samples with RNA integrity number (RIN) above 7, OD260/280: 2, and OD260/230 ≥ 2 were used. In addition, RNA degradation and contamination were monitored on 1% agarose gels before small RNA sequencing (sRNAseq).

### Library Preparation and Small RNA Sequencing

RNA quality was assessed using an Agilent TapeStation (Agilent, Palo Alto, CA, United States), and RNA concentration was quantified by Qubit 4.0 spectrophotometer. The library for sRNAseq was prepared using the Smarter smRNA-seq kit for Illumina (Takara Bio Inc., United States). The quantity and quality of amplified libraries were evaluated using Qubit (Invitrogen, Carlsbad, CA, United States) and Agilent TapeStation high sensitivity D1000 Screen Tape. sRNAseq libraries were sequenced using single-end 75 base pairs sequencing chemistry on NextSeq 500 instruments following the manufacturer’s protocols (Illumina).

### Real-Time Quantitative Reverse Transcription PCR

We isolated total RNA from EVs isolated from PB of independent cohort (astronauts S3, S13, S6) and PBMCs (astronauts S7, S8, S12, S22, S23, S24) at L-10 and R + 3. According to the manufacturer’s protocol, cDNA synthesis was performed with total RNA using the cDNA synthesis kit (Cat # 4368813, Applied BioSystems, Waltham, MA, United States). Real-time polymerase chain reaction (RT-qPCR) was performed using TaqMan probes (Master Mix, Cat #4444963, Applied BioSystems, Waltham, MA, United States) and QuantStudio™ three Real-Time PCR systems as recommended by the manufacturer. We measured the expression of the following snoRNAs indicated below.

**Table:** 

SNORA74A	Hs03298571_s1
SCARNA1	Hs03298705_s1
SNORA19	Hs03457523_s1
SNORA47	Hs03309497_s1
U6 snRNA	Forward Primer: 5′-CTCGCTTCGGCAGCACA
	Probe: 5′-ACGATACAGAGAAGATTAGCATGGCCC
	Reverse Primer: 5′-CGCTTCACGAATTTGCGTGTC

### Sequencing Data Analysis

Sequencing data were processed with Cutadapt (27) to remove adapter sequences, and read quality was assessed with FastQC^1^. Subsequently, reads were mapped with Bowtie (28) to the human genome (hg38), and gene count data were generated using featureCounts (29). Then, we used RUVSeq (30) to correct for batch effects and other unwanted variations. Differentially expressed genes were then identified with edgeR (31). Genes with false discovery rate adjusted *p*-value (FDR) < 0.05 and log_2_ fold change (FC) > 1 were considered significantly differentially expressed genes. Subsequently, we extracted differentially expressed snoRNAs and generated a heatmap using custom R scripts.

### 3D Structural Representation in Ribosomes

The cryo-EM atomic models of human 80S ribosomes (PDB ID: 6EKO) were used to represent modified nucleotides (32). Figures were generated using UCSF Chimera-X software^2^.

#### Statistical Analysis of qRT-PCR Results

Results are presented as mean ± standard error of the mean (SEM). Data were analyzed using an unpaired *t*-test for comparisons between means. Statistical analysis was performed using GraphPad Prism 9, version 9.2.0 (GraphPad Software, Inc., La Jolla, CA, United States). Differences were considered statistically significant at *p* < 0.05.

## Results

**FIGURE 1:**
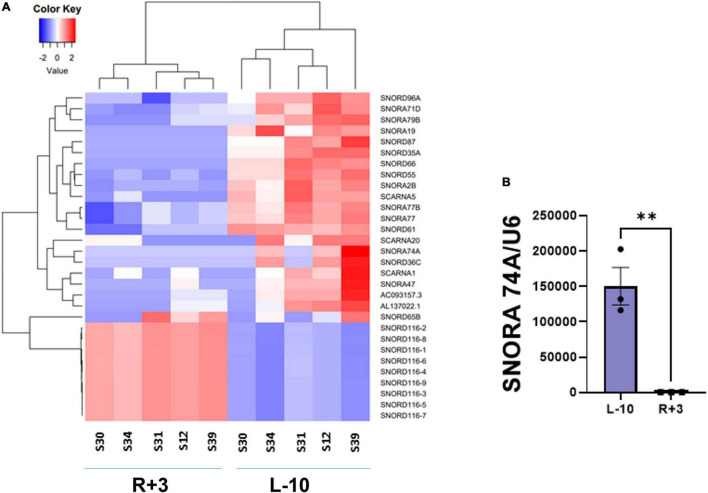
Small RNA sequencing of EV-derived RNA content. (A) Heat map of snoRNAs differentially expressed (fold change > 2 and FDR < 0.05) in the EVs derived from peripheral blood (PB) plasma collected 10 days before the launch (L-10) and 3 days after landing (R + 3) from five astronauts who flew Shuttle missions between 1998 and 2001 (*n* = 5 astronauts per group). (B) Validation of differentially expressed *SNORA74A* by RT-qPCR, normalized to *U6* snRNA, *n* = 3 astronauts/group. Data are presented as mean ± SEM, ^**^p < 0.01 vs. L-10 (two-sided unpaired students *t*-test).

**FIGURE 2:**
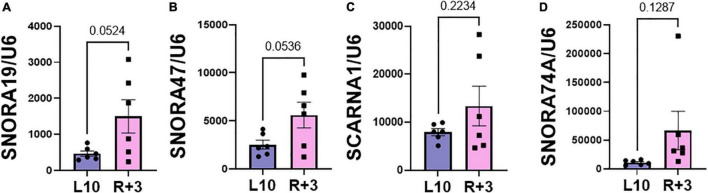
Validation of PBMC snoRNA by RT-qPCR. Validation of differentially expressed snoRNAs in the RNA from PB mononuclear cells (PBMCs) collected L-10 and R + 3 from six astronauts by RT-qPCR, normalized to *U6* snRNA (*n* = 6 astronauts/group), including (A) SNORA19, (B) SNORA47, (C) SCARNA1, (D) SNORA74A. Data are presented as mean ± SEM vs. L-10 (two-sided unpaired students *t*-test).

**FIGURE 3:**
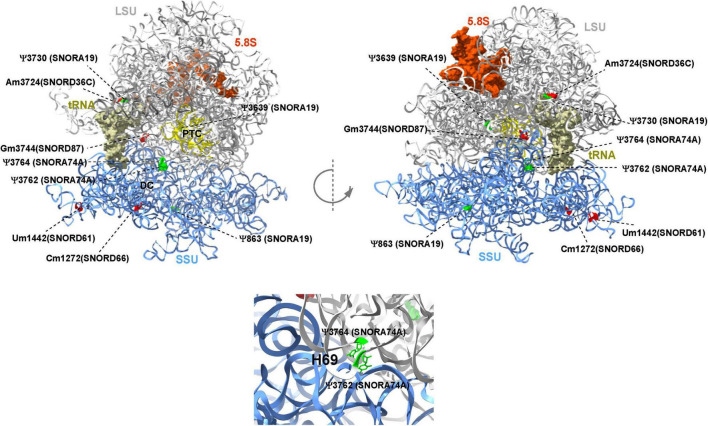
Deregulated snoRNAs guide specific rRNA modifications on human ribosome. Nm sites are indicated in red and ψ sites are indicated in green, respectively. The identity of snoRNA guiding each modification is shown. The peptidyl transferase center (PTC), E site tRNA, decoding center (DC), and helix H69 are indicated. The large subunit (LSU), small subunit (SSU), and 5.8S rRNA are colored in gray, blue, and brown, respectively. The 3D representation is based on the published CryoEM structure of the human 80S ribosome (PDB 6EK0) (32).

This report shows the existence of snoRNAs in the PB-EV of astronauts for the first time. After the quality and quantity of EVs were validated by NanoSight and exosome specific protein array as described previously (26), we further characterized the snoRNA profile in PB-EVs by sRNAseq. Computational analysis revealed differential expression of snoRNAs in the PB-EVs collected 10 days before mission launch (L-10) compared to 3 days after landing (R + 3) from five astronauts who flew short duration (median 12 days long) Shuttle missions between 1998 and 2001 (Figure 1A). The changes in PB-EV-derived snoRNA levels may be attributed to the expression of snoRNAs from the donor cells. Using stringent cutoff (FC > 2, FDR < 0.05 and *p* < 0.01), we detected 21 down-regulated snoRNAs and 9 upregulated snoRNAs in PB-EVs at R + 3 compared to L-10. Next, we assessed the top four down-regulated snoRNAs (SNORA74A, SCARNA1, SNORA19, and SNORA47) by RT-qPCR analysis in three independent astronauts (S3, S13, and S6). However, only SNORA74A was detectable in the L-10 PB-EVs, whereas SCARNA1, SNORA19, and SNORA47 levels were undetectable in R + 3 and L-10 PB-EVs. Consistent with the sRNAseq results, we found that SNORA74A was significantly down-regulated at R + 3 compared to L-10 (Figure 1B). Further, we validated the PCR products on 2% agarose gel and SNORA74A was undetectable in R + 3 compared to L-10 (Supplementary Figure 1). Given snoRNA modulation in the PB-EVs in the sRNAseq analysis, we speculated they could be detected in the astronauts’ PBMCs. Surprisingly, RT-qPCR analysis confirmed a significant increase in SNORA19, SNORA47, whereas SCARNA1 and SNOR74A remained unchanged at R + 3 (Figure 2). Given snoRNAs are known to guide 2′-*O*-methylation (Nm) or pseudouridine (ψ) on rRNA, snRNA, or mRNA (33), we further examined the post-transcriptional modifications that were affected. Remarkably, we observed that many of the downregulated snoRNAs guided 9 rRNA modifications, including four Nms and five ψs (Supplementary Table 1). Notably, some of these regulated snoRNAs guide adjacent positions in the rRNA, such as ψ3730*-*Am3724 and ψ3762*-*ψ3764, which may suggest the existence of coordinated regulation of rRNA modifications. We also observed that these snoRNAs guided modifications in critical functional domains of ribosomes, such as Cm1272 (in close proximity to the decoding center), ψ3762, and ψ3764 (in the helix-H69 inter-subunit domain) (Figure 3).

In summary, our study revealed for the first time dysregulation of snoRNAs in PB-EVs and PBMCs from astronauts, blood after a relatively short (median 12 days long) Shuttle missions and underscored the potential translational value of investigating snoRNAs as biomarkers for monitoring astronauts health.

## Discussion

This study examined the snoRNA content and space-travel associated changes in their expression in EVs derived from the peripheral blood of five astronauts who flew relatively short (median 12 days long) space Shuttle missions between 1998 and 2001. We provide the first report showing differential snoRNAs packaging in the astronauts’ plasma derived EVs pre- and post-flight.

Emerging evidence suggests snoRNA’s involvement in metabolism, diabetes, cancer, and heart development (20, 21, 23, 34). Specifically, dysregulation of SNORA47, SNORA74A, and SNORNA19 was previously described to be differentially expressed in different cancers (35–37). Further, SNORA74A was differentially expressed in the myocardium derived from infants with tetralogy of Fallot (38). In corroboration with these published studies, our data showed that SNORA47, SNORA74A, and SNORA19 were differentially expressed at 3 days post return from mission (R + 3) compared to baseline (L-10). These analyses provide a starting point for future studies to evaluate the predictive value of snoRNA packaging in PB-EVs or PBMCs in astronauts, who may be at risk of developing cancer or CVD.

Recent evidence has supported the notion that rRNA modifications such as Nm and Ψ guided by snoRNAs play an important role in translation by altering ribosome splicing and influencing snRNAs and mRNA stability by altering the binding of RNA-binding proteins (39, 40). In this study, we also observed that some of the dysregulated snoRNAs guide RNA modifications in critical functional domains of the ribosome, such as Cm1272 (in close proximity to the decoding center) and Ψ3762-Ψ3764 (in the helix-H69 inter-subunit domain). rRNA modification in these domains was previously suggested to be involved in translational regulation (41–43). Intriguingly, previous quantitative analysis of Nm suggested that Gm3744 (i.e., Gm3723) is highly variable in breast cancer patients (44). Notably, some of the differentially expressed snoRNAs identified in this study guide adjacent positions in the rRNA, such as Ψ3730-Am3724 and Ψ3762- Y3764, raising an intriguing coordinated regulation of rRNA modification. A more recent study demonstrated the contribution of a single rRNA modification toward generating ribosomes with specialized functions (34). Future functional studies are warranted to address the functional role of these specific RNA modifications in translation.

In summary, our study reports for the first time a dysregulation of snoRNAs in PB-EVs and PBMCs from astronauts’ blood after a relatively short (median 12 days long) Shuttle mission. Thus far, there is no reported evidence of CVD, cancer, or neurodegenerative diagnoses within this given astronaut cohort after approximately two decades. Albeit several limitations, the most considerable limitation of this study is the small sample size due to the rarity of access to astronauts’ PB; despite this limitation, this study demonstrates the feasibility of utilizing retrospectively collected and bio-banked samples for the assessment of EV snoRNA alterations. Furthermore, it highlights the need for further retrospective and prospective longitudinal studies paired with clinical data to assess the utility and validity of the potential translational value of snoRNAs as biomarkers to predict astronauts’ health and/or disease.

## Data Availability Statement

The datasets presented in this study can be found in online repositories. The names of the repository/repositories and accession number(s) can be found below: GEO, GSE200079.

## Ethics Statement

This study was approved by NASA and Icahn School of Medicine at Mount Sinai’s Institutional Review Board (STUDY00000075 and HSM19-00367, respectively). Written informed consent for participation was not required for this study in accordance with the national legislation and the institutional requirements.

## Author Contributions

DAG, AKR, and VNSG designed the research. AKR, MB, AB, AS, and AE performed the research. AKR, MB, AB, AS, AE, MC, and SU analyzed the data. DAG, AKR, KSR, MB, AB, LH, and VNSG wrote the manuscript. All authors approved the manuscript.

## Conflict of Interest

The authors declare that the research was conducted in the absence of any commercial or financial relationships that could be construed as a potential conflict of interest.

## Publisher’s Note

All claims expressed in this article are solely those of the authors and do not necessarily represent those of their affiliated organizations, or those of the publisher, the editors and the reviewers. Any product that may be evaluated in this article, or claim that may be made by its manufacturer, is not guaranteed or endorsed by the publisher.

## Footnotes

1
http://www.bioinformatics.babraham.ac.uk/projects/fastqc/

2
https://www.rbvi.ucsf.edu/chimerax/

## Supplementary Material

Supplementary Figure 1 Agarose gel electrophoresis of qPCR products. (A) SNORA74A PCR product was on run on 2% agarose gel (expected amplicon size: 69 nucleotides). (B) U6 snRNA was run on 2% agarose gel electrophoresis (expected amplicon size: 92 nucleotides).
