# Supplementary material for: Spaceflight-Associated Changes of snoRNAs in Peripheral Blood Mononuclear Cells and Plasma Exosomes—A Pilot Study
Source: Front Cardiovasc Med. 2022 Jun 24;9:886689. doi: 10.3389/fcvm.2022.886689 (PMC9267956; doi:10.3389/fcvm.2022.886689)
Supplement: Supplementary file 1 [file Table_1.docx]

| S.No | snoRNA | Location | snoRNA Type | Target RNA | Organization | Host gene |
| --- | --- | --- | --- | --- | --- | --- |
| 1 | [SNORD61 (U61)](http://snoopy.med.miyazaki-u.ac.jp/snorna_db.cgi?mode=sno_info&id=Homo_sapiens300644) | chrX:136879199-136879271 | C/D | 18S rRNA:U1442 | *Intronic* | [RBMX](http://snoopy.med.miyazaki-u.ac.jp/snorna_db.cgi?mode=code_seq_info&id=Homo_sapiens100456) |
| 2 | SNORD66  (HBII-142) | chr3:184325696-184325771 | C/D | 18S rRNA: C1272 | *Intronic* | EIF4G1 |
| 3 | SNORD87  (HBII-276) | chr8:66922467-66922555 | C/D | 28S rRNA: G3723 | *Mono* | Mono:295:AC011031.12 |
| 4 | SCARNA20 | chr17:60231516-60231646 | H/ACA | U12 snRNA-U28 |  |  |
| 6 | SNORD65B | chr8:41426655-41426727 | C/D | N/A | Mono | Mono:288; AC16868.8 |
| 7 | SNORD36C  (U36C) | chr9:133350847-133350912 | C/D | 28S rRNA: :A3703 | *Intronic* | [RPL7A](http://snoopy.med.miyazaki-u.ac.jp/snorna_db.cgi?mode=code_seq_info&id=Homo_sapiens100411) |
| 8 | [SNORA74A (U19)](http://snoopy.med.miyazaki-u.ac.jp/snorna_db.cgi?mode=sno_info&id=Homo_sapiens300551) | chr5:139278781-139278978 | H/ACA | 28S Rrna: U3741 and U3743,U3:U8 | *Intronic* | [MATR3](http://snoopy.med.miyazaki-u.ac.jp/snorna_db.cgi?mode=code_seq_info&id=Homo_sapiens100389) |
| 9 | [SCARNA1 (ACA35)](http://snoopy.med.miyazaki-u.ac.jp/snorna_db.cgi?mode=sno_info&id=Homo_sapiens300782) | chr1:27834401-27834566 | H/ACA | U2 snRNA: U89 | *Intronic* | [PPP1R8](http://snoopy.med.miyazaki-u.ac.jp/snorna_db.cgi?mode=code_seq_info&id=Homo_sapiens100542) |
| 10 | SNORA47  (HBI-115) | chr5:77080434-77080571 | H/ACA | 28S rRNA:U1766 | *Intronic* | Mono:SNORA47 |
| 11 | SNORD79  (ACA65) | chr4:184431966-184432049 | H/ACA | U6 snRNA: U31  U6 snRNA: U86 | Intronic | GTF2A1 |
| 12 | SNORA19  ACA19) | chr10:119060011-119060138 | H/ACA | [18S rRNA](http://snoopy.med.miyazaki-u.ac.jp/snorna_db.cgi?mode=target_info&id=Homo_sapiens900001): U863  [28S rRNA](http://snoopy.med.miyazaki-u.ac.jp/snorna_db.cgi?mode=target_info&id=Homo_sapiens900003)U3618 and U3709, | Intronic | [EIF3S10](http://snoopy.med.miyazaki-u.ac.jp/snorna_db.cgi?mode=code_seq_info&id=Homo_sapiens100342) |
| 13 | SNORD116-2  (HBII-85-2) | chr15:25054210-25054304 | C/D | Unknown | Poly | [hmm16326423](http://snoopy.med.miyazaki-u.ac.jp/snorna_db.cgi?mode=code_seq_info&id=Homo_sapiens100364) |
| 14 | SNORD116-1  (HBII-85-1) | chr15:25051477-25051571 | C/D | Unknown | Poly | [hmm16326423](http://snoopy.med.miyazaki-u.ac.jp/snorna_db.cgi?mode=code_seq_info&id=Homo_sapiens100364) |
| 15 | SNORD116-8  (HBII-85-8) | chr15:25070432-25070526 | C/D | Unknown | Intronic | [hmm16326423](http://snoopy.med.miyazaki-u.ac.jp/snorna_db.cgi?mode=code_seq_info&id=Homo_sapiens100364) |
| 16 | SNORD116-3  (HBII-85-3) | chr15:25056860-25056954 | C/D | Unknown | Poly | [hmm16326423](http://snoopy.med.miyazaki-u.ac.jp/snorna_db.cgi?mode=code_seq_info&id=Homo_sapiens100364) |
| 17 | SNORD116-5  (HBII-85-5) | chr15:25062333-25062427 | C/D | Unknown | Intronic | [hmm16326423](http://snoopy.med.miyazaki-u.ac.jp/snorna_db.cgi?mode=code_seq_info&id=Homo_sapiens100364) |
| 17 | SNORD116-9  (HBII-85-8) | chr15:25073107-25073201 | C/D | Unknown | Intronic | [hmm16326423](http://snoopy.med.miyazaki-u.ac.jp/snorna_db.cgi?mode=code_seq_info&id=Homo_sapiens100364) |
| 18 | SNORD116-7  (HBII-85-7) | chr15:25067788-25067882 | C/D | Unknown | Intronic | [hmm16326423](http://snoopy.med.miyazaki-u.ac.jp/snorna_db.cgi?mode=code_seq_info&id=Homo_sapiens100364) |
| 19 | SNORD116-6  (HBII-85-6) | chr15:25065026-25065121 | C/D | Unknown | Intronic | [hmm16326423](http://snoopy.med.miyazaki-u.ac.jp/snorna_db.cgi?mode=code_seq_info&id=Homo_sapiens100364) |
| 20 | SNORD116-4  (HBII-85-4) | chr15:25059538-25059633 | C/D | Unknown | Poly | [hmm16326423](http://snoopy.med.miyazaki-u.ac.jp/snorna_db.cgi?mode=code_seq_info&id=Homo_sapiens100364) |

**Supplementary table-1** Characteristics of differentially expressed snoRNAs. This table was prepared by using the following databases.

(Bouchard-Bourelle et al., 2020; Yoshihama, Nakao, & Kenmochi, 2013)

Bouchard-Bourelle, P., Desjardins-Henri, C., Mathurin-St-Pierre, D., Deschamps-Francoeur, G., Fafard-Couture, É., Garant, J.-M., . . . Scott, M. S. (2020). snoDB: an interactive database of human snoRNA sequences, abundance and interactions. *Nucleic acids research, 48*(D1), D220-D225.

Yoshihama, M., Nakao, A., & Kenmochi, N. (2013). snOPY: a small nucleolar RNA orthological gene database. *BMC research notes, 6*(1), 1-5.
