# Supplementary figures and images for: Spaceflight-Associated Changes of snoRNAs in Peripheral Blood Mononuclear Cells and Plasma Exosomes—A Pilot Study
Source: Front Cardiovasc Med. 2022 Jun 24;9:886689. doi: 10.3389/fcvm.2022.886689 (PMC9267956; doi:10.3389/fcvm.2022.886689)

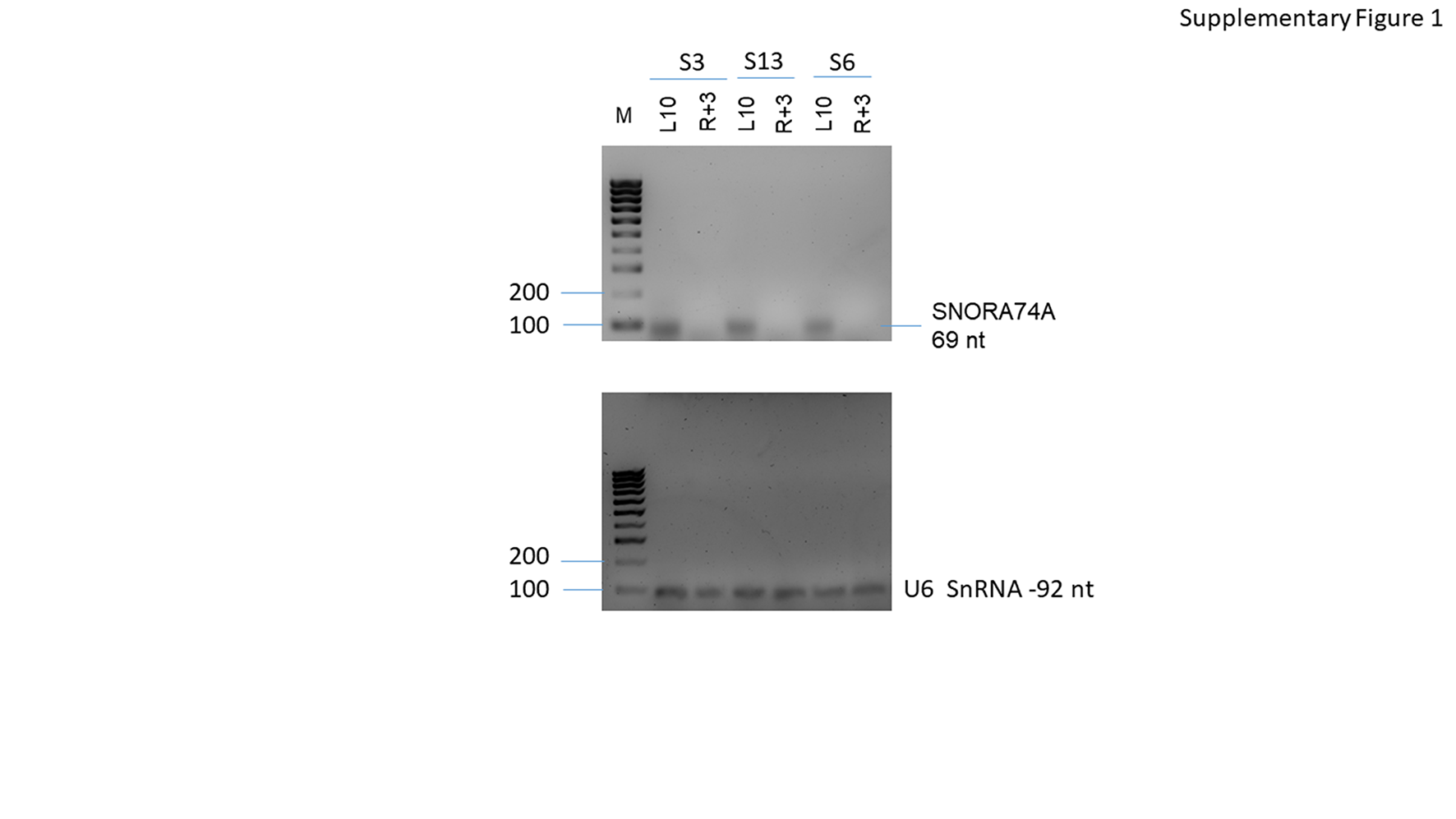

Supplement: Supplementary Figure 1 — Agarose gel electrophoresis of qPCR products. (A) SNORA74A PCR product was on run on 2% agarose gel (expected amplicon size: 69 nucleotides). (B) U6 snRNA was run on 2% agarose gel electrophoresis (expected amplicon size: 92 nucleotides). [file Image_1.TIF]
